# Air pollution and myocardial infarction in Poland

**DOI:** 10.1016/j.lanepe.2024.100933

**Published:** 2024-05-10

**Authors:** Mark R. Miller

**Affiliations:** Centre for Cardiovascular Science, University of Edinburgh, Edinburgh, UK

Air pollution is responsible for 6.7 million premature deaths annually.^1^ This is undoubtedly a huge underestimation given that this assessment does not encompass all air pollutants and is limited to a small subset of diseases. Cardiovascular disease accounts for approximately half of the mortality attributed to air pollution, and over the last decade studies have found associations between air pollution and all major cardiovascular diseases.^2^ In 2021 the World Health Organization (WHO) released new stringent guidelines for key air pollutants, to better reflect the evidence that even very low levels of air pollution have detrimental effects on health,^3^ although currently less than 10 countries meet these levels.^4^ In this issue of *Lancet Regional Health–Europe*, Kuźma and colleagues report associations between air pollution and hospitalizations for myocardial infarction in Eastern Poland.^5^

Eastern Poland has relatively high levels of air pollution for Europe, with episodes of “Polish smog” that is rich in particulate matter (PM) containing organic carbon species such as polyaromatic hydrocarbons (PAHs) which are considered to be some of the most harmful constituents of PM. The study distinguishes two categories of myocardial infarction: STEMI (S-T elevation myocardial infarction) and NSTEMI (non S-T elevation myocardial infarction), which in essence represent damage to the heart tissue with or without, respectively, occlusion of a major coronary artery leading to a prolonged and significant loss of blood flow to the cardiac tissue. While both are of concern, a STEMI carries a higher risk of life-threatening complications and requires emergency intervention to unblock the artery.

The authors found that the air pollutants PM_2.5_ (PM with a diameter of 2.5 μm for less) and nitrogen dioxide (NO_2_) increased the risk of an NSTEMI, whereas PM_2.5_ and sulphur dioxide (SO_2_) were more closely linked to increased risk of STEMI, with variations observed across lag periods, gender and age groups. Interestingly, the PAH benzo(a)pyrene was linked to both types of myocardial infarction, especially in rural populations (relative risk (RR) = 1.012, 95% CI: 1.005–1.018 in rural, RR = 1.008 CI: 1.002–1.015 in urban for NSTEMI; RR = 1.014, CI: 1.007–1.021 in rural, RR = 0.995 CI: 0.986–1.004 in urban for STEMI).

The study, using community level data (709 communities) from a large population with a mixed demographic (rural:urban and socioeconomic spread), builds on previous studies delineating the effects of air pollution on subtypes of myocardial infarction.^6^, ^7^, ^8^ Air pollution is far from a single entity–it is composed of thousands of different chemical species, each with different degrees of toxicity and distinct biological actions. SO_2_ is linked to sources such as coal burning and industrial emissions, whereas NO_2_ may arise principally from combustion processes like domestic heating and vehicle exhaust. The role for benzopyrene also suggests a key role for combustion sources in the observed cardiovascular effects, although more research is required to determine the direct toxicity induced by this pollutant at environmentally relevant levels.

Given the underlying pathology of a STEMI and NSTEMI are broadly the same, the mechanisms as to why certain air pollutants are more closely linked to specific cardiovascular exacerbations are not clear, but potentially could reflect the ability of air pollutants to increase blood clotting or impair fibrinolysis.^9^ Understanding these processes may offer insight into differences in cardiovascular disease presentation in different regions, and could be of value for emergency departments to prepare for acute coronary events resulting from high pollution episodes characterized by high levels of specific pollutants.

Kuźma et al. found greater risk ratios for air pollutants and STEMI in rural populations compared to urban. Many studies are weighted towards urban locations, because of the location of pollutant monitors and participant residences. This study highlights that air pollution is not just an urban issue, and that rural inhabitants are at risk too. A significant source of PM in this study will arise from domestic heating, however, PM can also be formed from ammonia emissions from agriculture. Many European countries have made progress in reducing PM, NO_2_ and SO_2_ emissions over the last few decades, however, less progress has been made with ammonia.^10^ Ozone–another key air pollutant for health, but not assessed in the current study–can also be higher in rural locations.

Understanding which pollutants drive which health impacts will be key in identifying which air pollution interventions that are the most effective (and cost-efficient) to protect health. Ultimately, multiple interventions will need to be employed together to significantly reduce air pollution, especially to reach the stringent targets set by the WHO. A step-change is needed in efforts to accelerate these ambitions. However, this is an objective worth striving for, as these targets are needed in order to curtail the substantial health effects of air pollution.

## Contributors

Mark Miller was the sole author and contributor of this article.

## Declaration of interests

The author has no conflicts of interest to declare.

## References

[1] World Health Organization The Global Health Observatory: air pollution data portal. https://www.who.int/data/gho/data/themes/air-pollution.

[2] Miller M.R., Newby D.E. (2020). Air pollution and cardiovascular disease: car sick. Cardiovasc Res.

[3] World Health Organization WHO global air quality guidelines: particulate matter (PM2.5 and PM10), ozone, nitrogen dioxide, sulfur dioxide and carbon monoxide. https://apps.who.int/iris/handle/10665/345329.

[4] IQAir 2023 World air quality report. https://www.iqair.com/gb/world-air-quality-report-press-kit.

[5] Kuźma Ł., Dąbrowski E., A K. (2024). Effect of air pollution exposure on risk of acute coronary syndromes in Poland: a nationwide population-based study (EP-PARTICLES study). Lancet Reg Health Europe.

[6] Gardner B., Ling F., Hopke P.K. (2014). Ambient fine particulate air pollution triggers ST-elevation myocardial infarction, but not non-ST elevation myocardial infarction: a case-crossover study. Part Fibre Toxicol.

[7] Pope C.A., Muhlestein J.B., Anderson J.L. (2015). Short-term exposure to fine particulate matter air pollution is preferentially associated with the risk of ST-segment elevation acute coronary events. J Am Heart Assoc.

[8] Butland B.K., Atkinson R.W., Milojevic A. (2016). Myocardial infarction, ST-elevation and non-ST-elevation myocardial infarction and modelled daily pollution concentrations: a case-crossover analysis of MINAP data. Open Heart.

[9] Robertson S., Miller M.R. (2018). Ambient air pollution and thrombosis. Part Fibre Toxicol.

[10] Fowler D., Brimblecombe P., Burrows J. (2020). A chronology of global air quality. Philos Trans A Math Phys Eng Sci.

